# Redetermination of the crystal structures of rare-earth trirhodium diboride *RE*Rh_3_B_2_ (*RE* = Pr, Nd and Sm) from single-crystal X-ray data

**DOI:** 10.1107/S2056989021013311

**Published:** 2022-01-01

**Authors:** Makoto Tokuda, Kunio Yubuta, Toetsu Shishido, Kazumasa Sugiyama

**Affiliations:** a Institute for Materials Research, Tohoku University, 2-1-1 Katahira, Aoba-ku, Sendai 980-8577, Japan

**Keywords:** single-crystal diffraction, crystal structure, boride, isotypism

## Abstract

The crystal structures of the rare-earth (*RE*) trirhodium diborides praseo­dymium trirhodium diboride, PrRh_3_B_2_, neodymium trirhodium diboride, NdRh_3_B_2_, and samarium trirhodium diboride, SmRh_3_B_2_, were refined on the basis of single-crystal X-ray diffraction data. The crystal chemistry of *RE*Rh_3_B_2_ (*RE*: Pr, Nd, and Sm) compounds has previously been analyzed mainly on the basis of powder samples [Ku *et al.* (1980 ▸). *Solid State Commun*. **35**, 91–96], and no structural investigation by single-crystal X-ray diffraction has been reported so far. The crystal structures of the three hexa­gonal *RE*Rh_3_B_2_ compounds are isotypic with that of CeRh_3_B_2_; *RE*, Rh and B sites are situated on special positions with site symmetry 6/*mmm* (Wyckoff position 1*a*), *mmm* (3*g*) and  *m*2 (2*c*), respectively. In comparison with the previous powder X-ray study of hexa­gonal *RE*Rh_3_B_2_, the present redetermination against single-crystal X-ray data has allowed for the modeling of all atoms with anisotropic displacement parameters (ADPs). The ADPs of the Rh atom in each of the structures result in an elongated displacement ellipsoid in the direction of the stacking of the Rh kagomé-type layer. The features of obtained ADPs of atoms are discussed in relation to *RE*Rh_3_B_2_-type and analogous structures.

## Abstract 2

The short *RE*⋯*RE* inter­atomic distance is a common structural feature of rare-earth trirhodium diborides *RE*Rh_3_B_2_ (Pr, Nd, and Sm). The crystal-structure redeterminations using single-crystal X-ray data revealed that the displacement ellipsoids of Rh and *RE* atoms elongated along the *c* axis are attributed to the unusually short *RE*⋯*RE* inter­atomic distances. Moreover, the anisotropic ellipsoids of Rh and *RE* could be associated with the appearance of disordered La_1–*x*_Rh_3_B_2_-type and/or Nd_1–*x*_Rh_*x*_Rh_3_B_2_-type structures.

## Chemical context

CeCo_3_B_2_-type *RE*Rh_3_B_2_ (*RE* = rare-earth element) compounds exhibit anomalous ferromagnetic properties (Malik *et al.*, 1983 ▸; Yamada *et al.*, 2004 ▸), and the unit-cell parameters of these compounds have been reported using powder X-ray diffraction (XRD) data (Ku *et al.*, 1980 ▸; Ku & Meisner, 1981 ▸). Higashi *et al.* (1987 ▸) analyzed the crystal structure of CeRh_3_B_2_ by using single-crystal XRD data and discussed the characteristics of the anisotropic atomic displacement parameters (ADP) of atoms in CeRh_3_B_2_ in relation to the structure. We report here the results of structural refinements using single crystals of *RE*Rh_3_B_2_ (*RE* = Pr, Nd, and Sm) grown by the arc-melting method.

## Structural commentary

**Figure 1:**
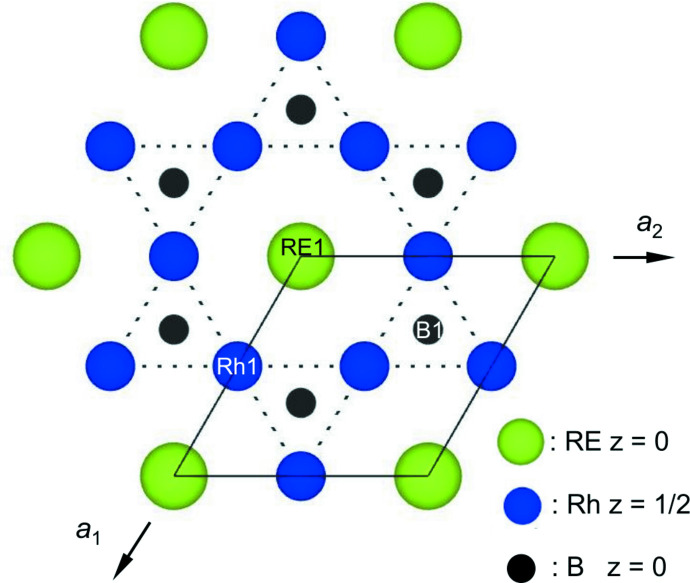
Structure of *RE*Rh_3_B_2_ compounds (space group: *P*6*/mmm*) as viewed along the *c* axis. B and *RE* atoms settle in the center of the trigonal and hexa­gonal prisms, respectively.

The crystal structures of hexa­gonal *RE*Rh_3_B_2_ (*RE*; La–Gd) compounds are isotypic with CeCo_3_B_2_ and crystallize in space-group type *P*6/*mmm* (Kuz’ma *et al.*, 1969 ▸). The CeCo_3_B_2_ type of structure is ordered and can be derived from the CaCu_5_ type of structure, whereby two distinct atoms (Rh and B) occupy the corresponding Cu sites. Each B atom is surrounded by six Rh atoms, forming a trigonal prism. Such [BRh_6_] trigonal prisms constitute a honeycomb structure and *RE* atoms are accommodated at the centers of the twelve [*RE*Rh_12_] hexa­gonal prisms, as shown in Fig. 1 ▸. The *RE*Rh_3_B_2_ type of structure can also be described as being built up of kagomé layers of Rh atoms stacked along the *c* axis with an *αα* stacking sequence and with B and *RE* atoms at the centers of the Rh triangular and hexa­gonal prisms, respectively.

**Figure 2:**
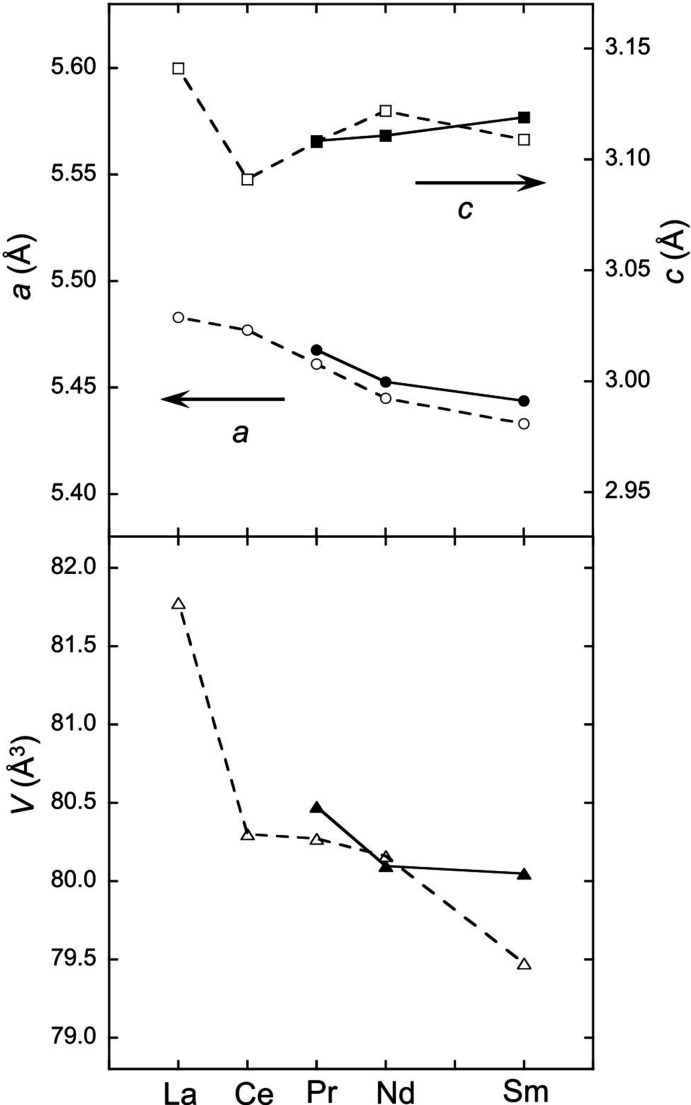
Unit-cell parameters *a* (circles), *c* (squares) and unit-cell volume (triangles) of *RE*Rh_3_B_2_ compounds. Closed and open marks refer to this study and previous work (Malik *et al.*, 1983 ▸), respectively.

The unit-cell parameters *a* and *c* and the unit-cell volume *V* of *RE*Rh_3_B_2_ (*RE* = La–Sm) compounds are shown in Fig. 2 ▸. The decrease in unit-cell volume results from the lanthanide contraction. The lattice parameters *a* and *c* decrease and increase, respectively. These anisotropic changes in the unit-cell parameters are consistent with those of a previous report using powder XRD analysis (Malik *et al.*, 1983 ▸).

The anisotropic change in the unit-cell parameters can be explained by the change in inter­atomic distances due to the lanthanide contraction. The ranges of B—Rh and *RE*—Rh distances are 2.2129 (1)–2.2151 (1) Å and 3.1370 (1)–3.1447 (1) Å (Table 1 ▸), respectively, which are close to the values of the sums of the atomic radii (*r*_Rh_ = 1.35 Å, *r*_B_ = 0.85 Å, *r*_Pr_ = 1.84 Å, *r*_Nd_ = 1.83 Å, and *r*_Sm_ = 1.81 Å; Daane *et al.*, 1954 ▸; Spedding *et al.*, 1956 ▸; Zachariasen, 1973 ▸). The *RE*—Rh inter­atomic distances decrease due to the effect of the lanthanoid contraction. Rh—Rh inter­atomic distances in the *ab* plane also decrease with a decrease in *RE*—Rh distances. By contrast, the Rh—Rh inter­atomic distances along the *c* axis increase. This causes the [*RE*Rh_12_] hexa­gonal and [BRh_6_] trigonal prisms to shrink horizontally and stretch vertically, resulting in decreases of the volumes of the hexa­gonal and trigonal prisms. Therefore, the unit-cells of *RE*Rh_3_B_2_ compounds change anisotropically, suggesting that the unit-cell changes elastically in response to the substitution of elements of different sizes at the *RE* site.

**Figure 3:**
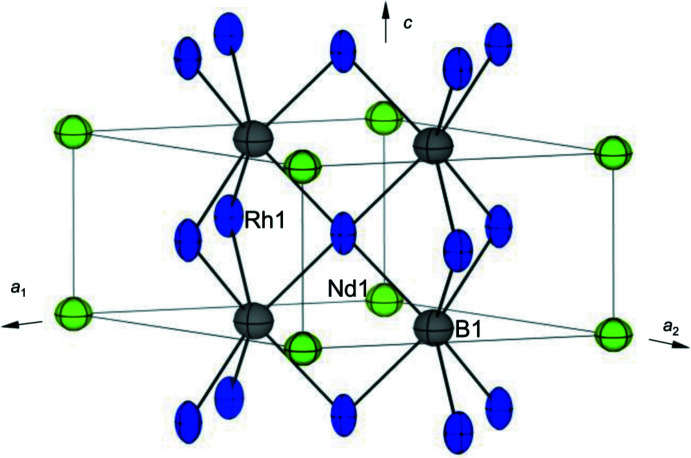
Displacement ellipsoids of each atom in NdRh_3_B_2_, with displacement ellipsoids drawn at the 99% probability level.

The obtained ADPs for each atom are summarized in Table 2 ▸. The displacement ellipsoid of the Rh atom shows a larger anisotropy than those of the B and *RE* atoms, as shown in Fig. 3 ▸. The *U*_33_ of Rh atoms is approximately 2.1–2.6 times larger than *U*_11_, which means that the displacement ellipsoids of Rh atoms are elongated along the *c* axis. The displacement ellipsoids of Rh atoms with large anisotropy correspond to the anisotropic electric resistivity of *RE*Rh_3_B_2_ compounds (Yamada *et al.*, 2004 ▸; Obiraki *et al.*, 2006 ▸). The ADPs of *RE* atoms are described as displacement ellipsoids suppressed in the *c* axis (*U*_11_ < *U*_33_). The feature of displacement ellipsoids of Rh and *RE* atoms is attributed to the unusually short *RE*—*RE* inter­atomic distances of 3.1084 (1)–3.1190 (1) Å, which are much shorter (15%) than the distance in the metal Pr, Nd, and Sm with hexa­gonal close-packed structures, (*i.e*., 3.67, 3.66, and 3.62 Å, respectively). The short *RE*—*RE* inter­atomic distance is a common feature of the CeCo_3_B_2_ type of structure. Anisotropy of electric or thermal conductivity is also expected to be observed in CeRh_3_B_2_ compounds.

The obtained anisotropic ADPs of each atom in the structures of *RE*Rh_3_B_2_ compounds can be discussed in terms of the nucleation of inter­stitial atoms or layers in PrRh_4.8_B_2_ (Higashi *et al.*, 1988 ▸). Higashi *et al.* (1988 ▸) discovered a new layered structure, namely, PrRh_4.8_B_2_, which is regarded as a stacking variant of a modified PrRh_3_B_2_ structure. The inter­stitial single Rh layer is positioned between the Rh kagomé layers of the modified PrRh_3_B_2_ blocks. The displacement ellipsoid in the stacking direction of the Rh atom in the PrRh_3_B_2_ structure implies that the Rh kagomé layer in PrRh_3_B_2_ could be a base for the nucleation of inter­stitial atoms or layers. The appearance of disordered La_1–*x*_Rh_3_B_2_ type and/or Nd_1–*x*_Rh_*x*_Rh_3_B_2_ type of structures (Ohtani *et al.*, 1983 ▸; Vlasse *et al.*, 1983 ▸; Ku *et al.*, 1985 ▸) might be associated with the anisotropic ADPs of Rh and *RE* atoms.

## Synthesis and crystallization

*RE*Rh_3_B_2_ (*RE* = Pr, Nd, and Sm) single crystals were grown using the arc-melting method. The starting materials used were *RE* elements (99.9%), along with Rh (99.95%), and B (99.5%). They were weighed at an atomic ratio of (*RE*+3Rh+2B), and the mixtures of the starting materials were placed in an argon-arc melting furnace (ACM-01, Diavac). Each product was remelted three times to improve homogeneity. The grown crystals were composed of homogeneous *RE*Rh_3_B_2_, and the atomic ratio Rh/*RE* was confirmed to be 3.00 by energy dispersive X-ray spectroscopy.

## Refinement details

Crystal data, data collection and structure refinement details are summarized in Table 3 ▸. A reciprocal space plot using all reflection data was in good agreement with the hexa­gonal lattice (*a* ≃ 5 Å and *c* ≃ 3 Å), and there was no evidence of superstructure reflections. The refinement was conducted under the assumption that the space group type was *P*6/*mmm*, as reported by Ku *et al.* (1980 ▸). Based on structural reports of La_1–*x*_Rh_3_B_2_ and Nd_1–*x*_Rh_*x*_Rh_3_B_2_, we determined whether Rh substitution and vacancies at the *RE* site were possible; however, the results were negative. Therefore, we concluded that the *RE* sites were completely occupied by *RE* elements. A correction for isotropic extinction was applied during the least-squares refinements. The final refinements were performed by applying anisotropic ADPs to each atom. The remaining electron densities located 0.7–0.6 Å around rhodium and *RE* heavy elements are censoring effects caused by the finite Fourier series.

## Supplementary Material

Crystal structure: contains datablock(s) global, I, II, III. DOI: 10.1107/S2056989021013311/wm5627sup1.cif

Structure factors: contains datablock(s) I. DOI: 10.1107/S2056989021013311/wm5627Isup5.hkl

Structure factors: contains datablock(s) II. DOI: 10.1107/S2056989021013311/wm5627IIsup6.hkl

Structure factors: contains datablock(s) III. DOI: 10.1107/S2056989021013311/wm5627IIIsup7.hkl

CCDC references: 2128890, 2128891, 2128892

Additional supporting information:  crystallographic information; 3D view; checkCIF report

## Figures and Tables

**Table 1:** Selected bond lengths (Å) in *RE*Rh_3_B_2_ (*RE* = Pr, Nd and Sm)

	PrRh_3_B_2_	NdRh_3_B_2_	SmRh_3_B_2_
*RE*—*RE* ×2	3.1084 (1)	3.1107 (1)	3.1190 (1)
*RE*—*RE* ×6	5.4676 (4)	5.4527 (3)	5.4438 (3)
*RE*—Rh ×12	3.1447 (1)	3.1388 (1)	3.1370 (1)
*RE*—B ×6	3.1557 (2)	3.1481 (1)	3.1430 (1)
B—Rh ×6	2.2151 (1)	2.2129 (1)	2.2140 (1)
B—B ×3	3.1084 (1)	3.1107 (1)	3.1190 (1)
B—B ×3	3.1567 (2)	3.1481 (1)	3.1430 (1)
Rh—Rh ×4	2.7338 (2)	2.7264 (1)	2.7219 (1)
Rh—Rh ×2	3.1084 (1)	3.1107 (1)	3.1190 (1)

**Table 2:** Atomic displacement parameters of *RE*, Rh, and B atoms in *RE*Rh_3_B_2_ (*RE* = Pr, Nd, and Sm)

Atom	*U*_11_ (Å^2^)	*U*_22_ (Å^2^)	*U*_33_ (Å^2^)	*U*_12_ (Å^2^)	*U*_eq_ (10 ^3^Å^2^)
*PrRh_3_B_2_*					
Pr	0.00861 (18)	0.00861	0.00780 (20)	0.00430 (9)	8.35 (1)
Rh	0.00495 (16)	0.00386 (18)	0.01040 (20)	0.00193 (9)	6.53 (1)
B	0.0095 (16)	0.0095	0.009 (2)	0.0048 (8)	9.3 (10)
*NdRh_3_B_2_*					
Nd	0.00896 (10)	0.00896	0.00662 (12)	0.00448 (5)	8.18 (9)
Rh	0.00492 (9)	0.00390 (11)	0.01104 (12)	0.00195 (5)	6.74 (8)
B	0.0100 (9)	0.0100	0.0079 (12)	0.0050 (4)	9.3 (6)
*SmRh_3_B_2_*					
Sm	0.00841 (12)	0.00841	0.00658 (14)	0.00420 (6)	7.80 (10)
Rh	0.00502 (11)	0.00389 (13)	0.01287 (14)	0.00194 (6)	7.39 (10)
B	0.0085 (10)	0.0085	0.0102 (15)	0.0043 (5)	9.1 (7)

**Table 3:** Experimental details

	PrRh_3_B_2_	NdRh_3_B_2_	SmRh_3_B_2_
Crystal data			
*M*_r_	471.26	474.59	480.70
Crystal system, space group	Hexagonal, *P*6/*m* *m* *m*	Hexagonal, *P*6/*m* *m* *m*	Hexagonal, *P*6/*m* *m* *m*
Temperature (K)	293	293	293
*a*, *c* (Å)	5.4676 (3), 3.10837 (16)	5.4527 (2), 3.11066 (13)	5.4438 (2), 3.11901 (12)
*V* (Å^3^)	80.47 (1)	80.10 (1)	80.05 (1)
*Z*	1	1	1
Radiation type	Mo *K*α	Mo *K*α	Mo *K*α
μ (mm^−1^)	29.43	30.57	32.61
Crystal size (mm)	0.05 × 0.03 × 0.03	0.05 × 0.05 × 0.02	0.06 × 0.05 × 0.02

Data collection			
Diffractometer	XtaLAB Synergy, Dualflex, HyPix	XtaLAB Synergy, Dualflex, HyPix	XtaLAB Synergy, Dualflex, HyPix
Absorption correction	Numerical (*CrysAlis PRO*; Rigaku OD, 2021 ▸)	Numerical (*CrysAlis PRO*; Rigaku OD, 2021 ▸)	Numerical (*CrysAlis PRO*; Rigaku OD, 2021 ▸)
*T*_min_, *T*_max_	0.423, 0.601	0.424, 0.611	0.324, 0.542
No. of measured, independent and observed [*I* > 2σ(*I*)] reflections	733, 131, 126	827, 131, 130	696, 129, 128
*R*_int_	0.017	0.010	0.011
(sin θ/λ)_max_ (Å^−1^)	0.909	0.908	0.907

Refinement			
*R*[*F* ^2^ > 2σ(*F* ^2^)], *wR*(*F* ^2^), *S*	0.018, 0.053, 1.21	0.012, 0.032, 1.15	0.012, 0.032, 1.13
No. of reflections	131	131	129
No. of parameters	8	8	9
Δρ_max_, Δρ_min_ (e Å^−3^)	1.80, −1.18	0.97, −2.46	1.76, −0.97

Computer programs: *CrysAlis PRO* (Rigaku OD, 2021 ▸), *SHELXT* (Sheldrick, 2015*a* ▸), *SHELXL* (Sheldrick, 2015*b* ▸), *VESTA* (Momma & Izumi, 2011 ▸) and *publCIF* (Westrip, 2010 ▸).

## supplementary crystallographic information

### Praseodymium trirhodium diboride (I) Crystal data

**Table:**

PrRh_3_B_2_	*D*_x_ = 9.724 Mg m^−^^3^
*M**_r_* = 471.26	Mo *K*α radiation, λ = 0.71073 Å
Hexagonal, *P*6/*m**m**m*	Cell parameters from 623 reflections
*a* = 5.4676 (3) Å	θ = 4.3–39.9°
*c* = 3.10837 (16) Å	µ = 29.43 mm^−^^1^
*V* = 80.47 (1) Å^3^	*T* = 293 K
*Z* = 1	Block, metallic
*F*(000) = 204	0.05 × 0.03 × 0.03 mm

### Praseodymium trirhodium diboride (I) Data collection

**Table:**

XtaLAB Synergy, Dualflex, HyPix diffractometer	131 independent reflections
Radiation source: micro-focus sealed X-ray tube, PhotonJet (Mo) X-ray Source	126 reflections with *I* > 2σ(*I*)
Mirror monochromator	*R*_int_ = 0.017
Detector resolution: 10.0000 pixels mm^-1^	θ_max_ = 40.3°, θ_min_ = 4.3°
ω scans	*h* = −9→7
Absorption correction: numerical (CrysAlisPro; Rigaku OD, 2021)	*k* = −9→9
*T*_min_ = 0.423, *T*_max_ = 0.601	*l* = −3→5
733 measured reflections	

### Praseodymium trirhodium diboride (I) Refinement

**Table:**

Refinement on *F*^2^	8 parameters
Least-squares matrix: full	0 restraints
*R*[*F*^2^ > 2σ(*F*^2^)] = 0.018	*w* = 1/[σ^2^(*F*_o_^2^) + (0.0347*P*)^2^] where *P* = (*F*_o_^2^ + 2*F*_c_^2^)/3
*wR*(*F*^2^) = 0.053	(Δ/σ)_max_ < 0.001
*S* = 1.21	Δρ_max_ = 1.80 e Å^−^^3^
131 reflections	Δρ_min_ = −1.18 e Å^−^^3^

### Praseodymium trirhodium diboride (I) Special details

**Table:**

Geometry. All esds (except the esd in the dihedral angle between two l.s. planes) are estimated using the full covariance matrix. The cell esds are taken into account individually in the estimation of esds in distances, angles and torsion angles; correlations between esds in cell parameters are only used when they are defined by crystal symmetry. An approximate (isotropic) treatment of cell esds is used for estimating esds involving l.s. planes.

### Praseodymium trirhodium diboride (I) Fractional atomic coordinates and isotropic or equivalent isotropic displacement parameters (Å^2^)

**Table:**

	*x*	*y*	*z*	*U*_iso_*/*U*_eq_	
Pr1	0.000000	0.000000	0.000000	0.00835 (14)	
Rh1	0.500000	0.000000	0.500000	0.00653 (13)	
B1	0.333333	0.666667	0.000000	0.0093 (10)	

### Praseodymium trirhodium diboride (I) Atomic displacement parameters (Å^2^)

**Table:**

	*U*^11^	*U*^22^	*U*^33^	*U*^12^	*U*^13^	*U*^23^
Pr1	0.00861 (18)	0.00861 (18)	0.0078 (2)	0.00430 (9)	0.000	0.000
Rh1	0.00495 (16)	0.00386 (18)	0.0104 (2)	0.00193 (9)	0.000	0.000
B1	0.0095 (16)	0.0095 (16)	0.009 (2)	0.0048 (8)	0.000	0.000

### Praseodymium trirhodium diboride (I) Geometric parameters (Å, º)

**Table:**

Pr1—Pr1^i^	3.1084 (2)	Pr1—Rh1^x^	3.1447 (1)
Pr1—Pr1^ii^	3.1084 (2)	Rh1—B1^xi^	2.2151 (1)
Pr1—Rh1^iii^	3.1447 (1)	Rh1—B1^xii^	2.2151 (1)
Pr1—Rh1^iv^	3.1447 (1)	Rh1—B1^xiii^	2.2151 (1)
Pr1—Rh1	3.1447 (1)	Rh1—B1^xiv^	2.2151 (1)
Pr1—Rh1^v^	3.1447 (1)	Rh1—Rh1^xv^	2.7338 (2)
Pr1—Rh1^vi^	3.1447 (1)	Rh1—Rh1^xvi^	2.7338 (2)
Pr1—Rh1^vii^	3.1447 (1)	Rh1—Rh1^iii^	2.7338 (2)
Pr1—Rh1^viii^	3.1447 (1)	Rh1—Rh1^xvii^	2.7338 (2)
Pr1—Rh1^ix^	3.1447 (1)	Rh1—Rh1^i^	3.1084 (2)
Pr1—Rh1^ii^	3.1447 (1)	Rh1—Rh1^ii^	3.1084 (2)
			
Pr1^i^—Pr1—Pr1^ii^	180.0	B1^xiii^—Rh1—Rh1^xv^	51.897 (2)
Pr1^i^—Pr1—Rh1^iii^	60.381 (1)	B1^xiv^—Rh1—Rh1^xv^	128.103 (2)
Pr1^ii^—Pr1—Rh1^iii^	119.619 (1)	B1^xi^—Rh1—Rh1^xvi^	128.103 (1)
Pr1^i^—Pr1—Rh1^iv^	119.619 (1)	B1^xii^—Rh1—Rh1^xvi^	51.897 (1)
Pr1^ii^—Pr1—Rh1^iv^	60.381 (1)	B1^xiii^—Rh1—Rh1^xvi^	128.103 (2)
Rh1^iii^—Pr1—Rh1^iv^	180.0	B1^xiv^—Rh1—Rh1^xvi^	51.897 (2)
Pr1^i^—Pr1—Rh1	60.381 (2)	Rh1^xv^—Rh1—Rh1^xvi^	180.0
Pr1^ii^—Pr1—Rh1	119.619 (2)	B1^xi^—Rh1—Rh1^iii^	51.896 (2)
Rh1^iii^—Pr1—Rh1	51.528 (1)	B1^xii^—Rh1—Rh1^iii^	128.104 (2)
Rh1^iv^—Pr1—Rh1	128.472 (1)	B1^xiii^—Rh1—Rh1^iii^	51.896 (2)
Pr1^i^—Pr1—Rh1^v^	119.619 (2)	B1^xiv^—Rh1—Rh1^iii^	128.104 (1)
Pr1^ii^—Pr1—Rh1^v^	60.381 (2)	Rh1^xv^—Rh1—Rh1^iii^	60.0
Rh1^iii^—Pr1—Rh1^v^	128.472 (1)	Rh1^xvi^—Rh1—Rh1^iii^	120.0
Rh1^iv^—Pr1—Rh1^v^	51.528 (1)	B1^xi^—Rh1—Rh1^xvii^	128.104 (2)
Rh1—Pr1—Rh1^v^	180.0	B1^xii^—Rh1—Rh1^xvii^	51.896 (2)
Pr1^i^—Pr1—Rh1^vi^	60.381 (2)	B1^xiii^—Rh1—Rh1^xvii^	128.104 (2)
Pr1^ii^—Pr1—Rh1^vi^	119.619 (2)	B1^xiv^—Rh1—Rh1^xvii^	51.896 (2)
Rh1^iii^—Pr1—Rh1^vi^	51.528 (1)	Rh1^xv^—Rh1—Rh1^xvii^	120.0
Rh1^iv^—Pr1—Rh1^vi^	128.472 (1)	Rh1^xvi^—Rh1—Rh1^xvii^	60.0
Rh1—Pr1—Rh1^vi^	97.678 (2)	Rh1^iii^—Rh1—Rh1^xvii^	180.0
Rh1^v^—Pr1—Rh1^vi^	82.322 (2)	B1^xi^—Rh1—Rh1^i^	45.442 (2)
Pr1^i^—Pr1—Rh1^vii^	119.619 (2)	B1^xii^—Rh1—Rh1^i^	134.558 (2)
Pr1^ii^—Pr1—Rh1^vii^	60.381 (2)	B1^xiii^—Rh1—Rh1^i^	134.558 (2)
Rh1^iii^—Pr1—Rh1^vii^	128.472 (1)	B1^xiv^—Rh1—Rh1^i^	45.442 (2)
Rh1^iv^—Pr1—Rh1^vii^	51.528 (1)	Rh1^xv^—Rh1—Rh1^i^	90.0
Rh1—Pr1—Rh1^vii^	82.322 (2)	Rh1^xvi^—Rh1—Rh1^i^	90.0
Rh1^v^—Pr1—Rh1^vii^	97.678 (2)	Rh1^iii^—Rh1—Rh1^i^	90.0
Rh1^vi^—Pr1—Rh1^vii^	180.0	Rh1^xvii^—Rh1—Rh1^i^	90.0
Pr1^i^—Pr1—Rh1^viii^	60.381 (2)	B1^xi^—Rh1—Rh1^ii^	134.558 (2)
Pr1^ii^—Pr1—Rh1^viii^	119.619 (2)	B1^xii^—Rh1—Rh1^ii^	45.442 (2)
Rh1^iii^—Pr1—Rh1^viii^	120.763 (4)	B1^xiii^—Rh1—Rh1^ii^	45.442 (2)
Rh1^iv^—Pr1—Rh1^viii^	59.238 (4)	B1^xiv^—Rh1—Rh1^ii^	134.558 (2)
Rh1—Pr1—Rh1^viii^	97.678 (3)	Rh1^xv^—Rh1—Rh1^ii^	90.0
Rh1^v^—Pr1—Rh1^viii^	82.322 (3)	Rh1^xvi^—Rh1—Rh1^ii^	90.0
Rh1^vi^—Pr1—Rh1^viii^	97.678 (3)	Rh1^iii^—Rh1—Rh1^ii^	90.0
Rh1^vii^—Pr1—Rh1^viii^	82.322 (3)	Rh1^xvii^—Rh1—Rh1^ii^	90.0
Pr1^i^—Pr1—Rh1^ix^	119.619 (2)	Rh1^i^—Rh1—Rh1^ii^	180.0
Pr1^ii^—Pr1—Rh1^ix^	60.381 (2)	B1^xi^—Rh1—Pr1	110.289 (2)
Rh1^iii^—Pr1—Rh1^ix^	59.238 (4)	B1^xii^—Rh1—Pr1	69.711 (3)
Rh1^iv^—Pr1—Rh1^ix^	120.763 (4)	B1^xiii^—Rh1—Pr1	69.710 (2)
Rh1—Pr1—Rh1^ix^	82.322 (3)	B1^xiv^—Rh1—Pr1	110.290 (2)
Rh1^v^—Pr1—Rh1^ix^	97.678 (3)	Rh1^xv^—Rh1—Pr1	115.764 (1)
Rh1^vi^—Pr1—Rh1^ix^	82.322 (3)	Rh1^xvi^—Rh1—Pr1	64.236 (1)
Rh1^vii^—Pr1—Rh1^ix^	97.678 (3)	Rh1^iii^—Rh1—Pr1	64.236 (1)
Rh1^viii^—Pr1—Rh1^ix^	180.0	Rh1^xvii^—Rh1—Pr1	115.764 (1)
Pr1^i^—Pr1—Rh1^ii^	119.619 (2)	Rh1^i^—Rh1—Pr1	119.619 (1)
Pr1^ii^—Pr1—Rh1^ii^	60.381 (2)	Rh1^ii^—Rh1—Pr1	60.381 (2)
Rh1^iii^—Pr1—Rh1^ii^	82.322 (3)	B1^xi^—Rh1—Pr1^xviii^	69.711 (2)
Rh1^iv^—Pr1—Rh1^ii^	97.678 (3)	B1^xii^—Rh1—Pr1^xviii^	110.289 (3)
Rh1—Pr1—Rh1^ii^	59.238 (4)	B1^xiii^—Rh1—Pr1^xviii^	110.290 (2)
Rh1^v^—Pr1—Rh1^ii^	120.762 (4)	B1^xiv^—Rh1—Pr1^xviii^	69.710 (2)
Rh1^vi^—Pr1—Rh1^ii^	128.472 (1)	Rh1^xv^—Rh1—Pr1^xviii^	64.236 (1)
Rh1^vii^—Pr1—Rh1^ii^	51.528 (1)	Rh1^xvi^—Rh1—Pr1^xviii^	115.764 (1)
Rh1^viii^—Pr1—Rh1^ii^	128.472 (1)	Rh1^iii^—Rh1—Pr1^xviii^	115.764 (1)
Rh1^ix^—Pr1—Rh1^ii^	51.528 (1)	Rh1^xvii^—Rh1—Pr1^xviii^	64.236 (1)
Pr1^i^—Pr1—Rh1^x^	60.381 (2)	Rh1^i^—Rh1—Pr1^xviii^	60.381 (2)
Pr1^ii^—Pr1—Rh1^x^	119.619 (2)	Rh1^ii^—Rh1—Pr1^xviii^	119.619 (2)
Rh1^iii^—Pr1—Rh1^x^	97.678 (3)	Pr1—Rh1—Pr1^xviii^	180.0
Rh1^iv^—Pr1—Rh1^x^	82.322 (3)	Rh1^xix^—B1—Rh1^xx^	138.257 (2)
Rh1—Pr1—Rh1^x^	120.762 (4)	Rh1^xix^—B1—Rh1^vi^	89.116 (4)
Rh1^v^—Pr1—Rh1^x^	59.238 (4)	Rh1^xx^—B1—Rh1^vi^	76.206 (4)
Rh1^vi^—Pr1—Rh1^x^	51.528 (1)	Rh1^xix^—B1—Rh1^xxi^	76.206 (4)
Rh1^vii^—Pr1—Rh1^x^	128.472 (1)	Rh1^xx^—B1—Rh1^xxi^	89.116 (4)
Rh1^viii^—Pr1—Rh1^x^	51.528 (1)	Rh1^vi^—B1—Rh1^xxi^	138.257 (2)
Rh1^ix^—Pr1—Rh1^x^	128.472 (1)	Rh1^xix^—B1—Rh1^iii^	138.257 (2)
Rh1^ii^—Pr1—Rh1^x^	180.0	Rh1^xx^—B1—Rh1^iii^	76.206 (4)
B1^xi^—Rh1—B1^xii^	180.0	Rh1^vi^—B1—Rh1^iii^	76.206 (4)
B1^xi^—Rh1—B1^xiii^	89.116 (4)	Rh1^xxi^—B1—Rh1^iii^	138.257 (2)
B1^xii^—Rh1—B1^xiii^	90.884 (4)	Rh1^xix^—B1—Rh1^ix^	76.206 (4)
B1^xi^—Rh1—B1^xiv^	90.884 (4)	Rh1^xx^—B1—Rh1^ix^	138.257 (2)
B1^xii^—Rh1—B1^xiv^	89.116 (4)	Rh1^vi^—B1—Rh1^ix^	138.257 (2)
B1^xiii^—Rh1—B1^xiv^	180.0	Rh1^xxi^—B1—Rh1^ix^	76.206 (4)
B1^xi^—Rh1—Rh1^xv^	51.897 (1)	Rh1^iii^—B1—Rh1^ix^	89.116 (4)
B1^xii^—Rh1—Rh1^xv^	128.103 (1)		

Symmetry codes: (i) *x*, *y*, *z*+1; (ii) *x*, *y*, *z*−1; (iii) −*x*+*y*+1, −*x*+1, *z*; (iv) −*x*+*y*, −*x*, *z*−1; (v) *x*−1, *y*, *z*−1; (vi) −*y*, *x*−*y*, *z*; (vii) −*y*, *x*−*y*−1, *z*−1; (viii) −*x*+*y*, −*x*, *z*; (ix) −*x*+*y*+1, −*x*+1, *z*−1; (x) *x*−1, *y*, *z*; (xi) −*x*+1, −*y*+1, −*z*+1; (xii) *x*, *y*−1, *z*; (xiii) −*x*+1, −*y*+1, −*z*; (xiv) *x*, *y*−1, *z*+1; (xv) −*y*+1, *x*−*y*, *z*; (xvi) −*y*, *x*−*y*−1, *z*; (xvii) −*x*+*y*+1, −*x*, *z*; (xviii) *x*+1, *y*, *z*+1; (xix) −*y*, *x*−*y*, *z*−1; (xx) *x*, *y*+1, *z*; (xxi) *x*, *y*+1, *z*−1.

### Neodymium trirhodium diboride (II) Crystal data

**Table:**

NdRh_3_B_2_	*D*_x_ = 9.839 Mg m^−^^3^
*M**_r_* = 474.59	Mo *K*α radiation, λ = 0.71073 Å
Hexagonal, *P*6/*m**m**m*	Cell parameters from 729 reflections
*a* = 5.4527 (2) Å	θ = 4.3–40.7°
*c* = 3.11066 (13) Å	µ = 30.57 mm^−^^1^
*V* = 80.10 (1) Å^3^	*T* = 293 K
*Z* = 1	Block, metallic
*F*(000) = 205	0.05 × 0.05 × 0.02 mm

### Neodymium trirhodium diboride (II) Data collection

**Table:**

XtaLAB Synergy, Dualflex, HyPix diffractometer	131 independent reflections
Radiation source: micro-focus sealed X-ray tube, PhotonJet (Mo) X-ray Source	130 reflections with *I* > 2σ(*I*)
Mirror monochromator	*R*_int_ = 0.010
Detector resolution: 10.0000 pixels mm^-1^	θ_max_ = 40.2°, θ_min_ = 4.3°
ω scans	*h* = −9→8
Absorption correction: numerical (CrysAlisPro; Rigaku OD, 2021)	*k* = −7→9
*T*_min_ = 0.424, *T*_max_ = 0.611	*l* = −3→5
827 measured reflections	

### Neodymium trirhodium diboride (II) Refinement

**Table:**

Refinement on *F*^2^	8 parameters
Least-squares matrix: full	0 restraints
*R*[*F*^2^ > 2σ(*F*^2^)] = 0.012	*w* = 1/[σ^2^(*F*_o_^2^) + (0.022*P*)^2^] where *P* = (*F*_o_^2^ + 2*F*_c_^2^)/3
*wR*(*F*^2^) = 0.032	(Δ/σ)_max_ < 0.001
*S* = 1.15	Δρ_max_ = 0.97 e Å^−^^3^
131 reflections	Δρ_min_ = −2.46 e Å^−^^3^

### Neodymium trirhodium diboride (II) Special details

**Table:**

Geometry. All esds (except the esd in the dihedral angle between two l.s. planes) are estimated using the full covariance matrix. The cell esds are taken into account individually in the estimation of esds in distances, angles and torsion angles; correlations between esds in cell parameters are only used when they are defined by crystal symmetry. An approximate (isotropic) treatment of cell esds is used for estimating esds involving l.s. planes.

### Neodymium trirhodium diboride (II) Fractional atomic coordinates and isotropic or equivalent isotropic displacement parameters (Å^2^)

**Table:**

	*x*	*y*	*z*	*U*_iso_*/*U*_eq_	
Nd1	0.000000	0.000000	0.000000	0.00818 (9)	
Rh1	0.500000	0.000000	0.500000	0.00674 (8)	
B1	0.333333	0.666667	0.000000	0.0093 (6)	

### Neodymium trirhodium diboride (II) Atomic displacement parameters (Å^2^)

**Table:**

	*U*^11^	*U*^22^	*U*^33^	*U*^12^	*U*^13^	*U*^23^
Nd1	0.00896 (10)	0.00896 (10)	0.00662 (12)	0.00448 (5)	0.000	0.000
Rh1	0.00492 (9)	0.00390 (11)	0.01104 (12)	0.00195 (5)	0.000	0.000
B1	0.0100 (9)	0.0100 (9)	0.0079 (12)	0.0050 (4)	0.000	0.000

### Neodymium trirhodium diboride (II) Geometric parameters (Å, º)

**Table:**

Nd1—Nd1^i^	3.1107 (1)	Nd1—Rh1^ii^	3.1388 (1)
Nd1—Nd1^ii^	3.1107 (1)	Rh1—B1^xi^	2.2129 (1)
Nd1—Rh1^iii^	3.1388 (1)	Rh1—B1^xii^	2.2129 (1)
Nd1—Rh1^iv^	3.1388 (1)	Rh1—B1^xiii^	2.2129 (1)
Nd1—Rh1^v^	3.1388 (1)	Rh1—B1^xiv^	2.2129 (1)
Nd1—Rh1^vi^	3.1388 (1)	Rh1—Rh1^xv^	2.7264 (1)
Nd1—Rh1	3.1388 (1)	Rh1—Rh1^xvi^	2.7264 (1)
Nd1—Rh1^vii^	3.1388 (1)	Rh1—Rh1^iii^	2.7264 (1)
Nd1—Rh1^viii^	3.1388 (1)	Rh1—Rh1^xvii^	2.7264 (1)
Nd1—Rh1^ix^	3.1388 (1)	Rh1—Rh1^i^	3.1107 (1)
Nd1—Rh1^x^	3.1388 (1)	Rh1—Rh1^ii^	3.1107 (1)
			
Nd1^i^—Nd1—Nd1^ii^	180.0	B1^xiii^—Rh1—Rh1^xv^	51.974 (1)
Nd1^i^—Nd1—Rh1^iii^	60.296 (1)	B1^xiv^—Rh1—Rh1^xv^	128.026 (1)
Nd1^ii^—Nd1—Rh1^iii^	119.704 (1)	B1^xi^—Rh1—Rh1^xvi^	128.026 (1)
Nd1^i^—Nd1—Rh1^iv^	119.704 (1)	B1^xii^—Rh1—Rh1^xvi^	51.974 (1)
Nd1^ii^—Nd1—Rh1^iv^	60.296 (1)	B1^xiii^—Rh1—Rh1^xvi^	128.026 (2)
Rh1^iii^—Nd1—Rh1^iv^	180.0	B1^xiv^—Rh1—Rh1^xvi^	51.974 (1)
Nd1^i^—Nd1—Rh1^v^	60.296 (1)	Rh1^xv^—Rh1—Rh1^xvi^	180.0
Nd1^ii^—Nd1—Rh1^v^	119.704 (1)	B1^xi^—Rh1—Rh1^iii^	51.973 (1)
Rh1^iii^—Nd1—Rh1^v^	51.481 (1)	B1^xii^—Rh1—Rh1^iii^	128.027 (1)
Rh1^iv^—Nd1—Rh1^v^	128.519 (1)	B1^xiii^—Rh1—Rh1^iii^	51.973 (1)
Nd1^i^—Nd1—Rh1^vi^	119.704 (1)	B1^xiv^—Rh1—Rh1^iii^	128.027 (1)
Nd1^ii^—Nd1—Rh1^vi^	60.296 (1)	Rh1^xv^—Rh1—Rh1^iii^	60.0
Rh1^iii^—Nd1—Rh1^vi^	128.519 (1)	Rh1^xvi^—Rh1—Rh1^iii^	120.0
Rh1^iv^—Nd1—Rh1^vi^	51.481 (1)	B1^xi^—Rh1—Rh1^xvii^	128.027 (2)
Rh1^v^—Nd1—Rh1^vi^	180.0	B1^xii^—Rh1—Rh1^xvii^	51.973 (1)
Nd1^i^—Nd1—Rh1	60.296 (1)	B1^xiii^—Rh1—Rh1^xvii^	128.027 (1)
Nd1^ii^—Nd1—Rh1	119.704 (1)	B1^xiv^—Rh1—Rh1^xvii^	51.973 (1)
Rh1^iii^—Nd1—Rh1	51.481 (1)	Rh1^xv^—Rh1—Rh1^xvii^	120.0
Rh1^iv^—Nd1—Rh1	128.519 (1)	Rh1^xvi^—Rh1—Rh1^xvii^	60.0
Rh1^v^—Nd1—Rh1	97.568 (2)	Rh1^iii^—Rh1—Rh1^xvii^	180.0
Rh1^vi^—Nd1—Rh1	82.432 (2)	B1^xi^—Rh1—Rh1^i^	45.343 (2)
Nd1^i^—Nd1—Rh1^vii^	119.704 (1)	B1^xii^—Rh1—Rh1^i^	134.657 (1)
Nd1^ii^—Nd1—Rh1^vii^	60.296 (1)	B1^xiii^—Rh1—Rh1^i^	134.657 (2)
Rh1^iii^—Nd1—Rh1^vii^	128.519 (1)	B1^xiv^—Rh1—Rh1^i^	45.343 (2)
Rh1^iv^—Nd1—Rh1^vii^	51.481 (1)	Rh1^xv^—Rh1—Rh1^i^	90.0
Rh1^v^—Nd1—Rh1^vii^	82.432 (2)	Rh1^xvi^—Rh1—Rh1^i^	90.0
Rh1^vi^—Nd1—Rh1^vii^	97.568 (2)	Rh1^iii^—Rh1—Rh1^i^	90.0
Rh1—Nd1—Rh1^vii^	180.0	Rh1^xvii^—Rh1—Rh1^i^	90.0
Nd1^i^—Nd1—Rh1^viii^	60.296 (2)	B1^xi^—Rh1—Rh1^ii^	134.657 (2)
Nd1^ii^—Nd1—Rh1^viii^	119.704 (2)	B1^xii^—Rh1—Rh1^ii^	45.343 (2)
Rh1^iii^—Nd1—Rh1^viii^	120.592 (3)	B1^xiii^—Rh1—Rh1^ii^	45.343 (2)
Rh1^iv^—Nd1—Rh1^viii^	59.408 (3)	B1^xiv^—Rh1—Rh1^ii^	134.657 (2)
Rh1^v^—Nd1—Rh1^viii^	97.568 (2)	Rh1^xv^—Rh1—Rh1^ii^	90.0
Rh1^vi^—Nd1—Rh1^viii^	82.432 (2)	Rh1^xvi^—Rh1—Rh1^ii^	90.0
Rh1—Nd1—Rh1^viii^	97.568 (2)	Rh1^iii^—Rh1—Rh1^ii^	90.0
Rh1^vii^—Nd1—Rh1^viii^	82.432 (2)	Rh1^xvii^—Rh1—Rh1^ii^	90.0
Nd1^i^—Nd1—Rh1^ix^	119.704 (2)	Rh1^i^—Rh1—Rh1^ii^	180.0
Nd1^ii^—Nd1—Rh1^ix^	60.296 (2)	B1^xi^—Rh1—Nd1	110.381 (2)
Rh1^iii^—Nd1—Rh1^ix^	59.408 (3)	B1^xii^—Rh1—Nd1	69.619 (2)
Rh1^iv^—Nd1—Rh1^ix^	120.592 (3)	B1^xiii^—Rh1—Nd1	69.617 (2)
Rh1^v^—Nd1—Rh1^ix^	82.432 (2)	B1^xiv^—Rh1—Nd1	110.383 (2)
Rh1^vi^—Nd1—Rh1^ix^	97.568 (2)	Rh1^xv^—Rh1—Nd1	115.7
Rh1—Nd1—Rh1^ix^	82.432 (2)	Rh1^xvi^—Rh1—Nd1	64.3
Rh1^vii^—Nd1—Rh1^ix^	97.568 (2)	Rh1^iii^—Rh1—Nd1	64.259 (1)
Rh1^viii^—Nd1—Rh1^ix^	180.0	Rh1^xvii^—Rh1—Nd1	115.741 (1)
Nd1^i^—Nd1—Rh1^x^	60.296 (1)	Rh1^i^—Rh1—Nd1	119.704 (1)
Nd1^ii^—Nd1—Rh1^x^	119.704 (1)	Rh1^ii^—Rh1—Nd1	60.296 (1)
Rh1^iii^—Nd1—Rh1^x^	97.568 (3)	B1^xi^—Rh1—Nd1^xviii^	69.619 (2)
Rh1^iv^—Nd1—Rh1^x^	82.432 (3)	B1^xii^—Rh1—Nd1^xviii^	110.381 (2)
Rh1^v^—Nd1—Rh1^x^	51.481 (1)	B1^xiii^—Rh1—Nd1^xviii^	110.383 (2)
Rh1^vi^—Nd1—Rh1^x^	128.519 (1)	B1^xiv^—Rh1—Nd1^xviii^	69.617 (2)
Rh1—Nd1—Rh1^x^	120.592 (3)	Rh1^xv^—Rh1—Nd1^xviii^	64.3
Rh1^vii^—Nd1—Rh1^x^	59.408 (3)	Rh1^xvi^—Rh1—Nd1^xviii^	115.7
Rh1^viii^—Nd1—Rh1^x^	51.481 (1)	Rh1^iii^—Rh1—Nd1^xviii^	115.741 (1)
Rh1^ix^—Nd1—Rh1^x^	128.519 (1)	Rh1^xvii^—Rh1—Nd1^xviii^	64.259 (1)
Nd1^i^—Nd1—Rh1^ii^	119.704 (1)	Rh1^i^—Rh1—Nd1^xviii^	60.296 (1)
Nd1^ii^—Nd1—Rh1^ii^	60.296 (1)	Rh1^ii^—Rh1—Nd1^xviii^	119.704 (1)
Rh1^iii^—Nd1—Rh1^ii^	82.432 (3)	Nd1—Rh1—Nd1^xviii^	180.0
Rh1^iv^—Nd1—Rh1^ii^	97.568 (3)	Rh1^xix^—B1—Rh1^xx^	138.332 (1)
Rh1^v^—Nd1—Rh1^ii^	128.519 (1)	Rh1^xix^—B1—Rh1^v^	89.314 (4)
Rh1^vi^—Nd1—Rh1^ii^	51.481 (1)	Rh1^xx^—B1—Rh1^v^	76.053 (3)
Rh1—Nd1—Rh1^ii^	59.408 (3)	Rh1^xix^—B1—Rh1^xxi^	76.053 (3)
Rh1^vii^—Nd1—Rh1^ii^	120.592 (3)	Rh1^xx^—B1—Rh1^xxi^	89.314 (4)
Rh1^viii^—Nd1—Rh1^ii^	128.519 (1)	Rh1^v^—B1—Rh1^xxi^	138.332 (1)
Rh1^ix^—Nd1—Rh1^ii^	51.481 (1)	Rh1^xix^—B1—Rh1^iii^	138.332 (1)
Rh1^x^—Nd1—Rh1^ii^	180.0	Rh1^xx^—B1—Rh1^iii^	76.053 (2)
B1^xi^—Rh1—B1^xii^	180.0	Rh1^v^—B1—Rh1^iii^	76.053 (3)
B1^xi^—Rh1—B1^xiii^	89.314 (4)	Rh1^xxi^—B1—Rh1^iii^	138.332 (1)
B1^xii^—Rh1—B1^xiii^	90.686 (4)	Rh1^xix^—B1—Rh1^ix^	76.053 (3)
B1^xi^—Rh1—B1^xiv^	90.686 (4)	Rh1^xx^—B1—Rh1^ix^	138.332 (1)
B1^xii^—Rh1—B1^xiv^	89.314 (4)	Rh1^v^—B1—Rh1^ix^	138.332 (2)
B1^xiii^—Rh1—B1^xiv^	180.0	Rh1^xxi^—B1—Rh1^ix^	76.053 (3)
B1^xi^—Rh1—Rh1^xv^	51.974 (1)	Rh1^iii^—B1—Rh1^ix^	89.314 (4)
B1^xii^—Rh1—Rh1^xv^	128.026 (1)		

Symmetry codes: (i) *x*, *y*, *z*+1; (ii) *x*, *y*, *z*−1; (iii) −*x*+*y*+1, −*x*+1, *z*; (iv) −*x*+*y*, −*x*, *z*−1; (v) −*y*, *x*−*y*, *z*; (vi) −*y*, *x*−*y*−1, *z*−1; (vii) *x*−1, *y*, *z*−1; (viii) −*x*+*y*, −*x*, *z*; (ix) −*x*+*y*+1, −*x*+1, *z*−1; (x) *x*−1, *y*, *z*; (xi) −*x*+1, −*y*+1, −*z*+1; (xii) *x*, *y*−1, *z*; (xiii) −*x*+1, −*y*+1, −*z*; (xiv) *x*, *y*−1, *z*+1; (xv) −*y*+1, *x*−*y*, *z*; (xvi) −*y*, *x*−*y*−1, *z*; (xvii) −*x*+*y*+1, −*x*, *z*; (xviii) *x*+1, *y*, *z*+1; (xix) −*y*, *x*−*y*, *z*−1; (xx) *x*, *y*+1, *z*; (xxi) *x*, *y*+1, *z*−1.

### Samarium trirhodium diboride (III) Crystal data

**Table:**

SmRh_3_B_2_	*D*_x_ = 9.972 Mg m^−^^3^
*M**_r_* = 480.70	Mo *K*α radiation, λ = 0.71073 Å
Hexagonal, *P*6/*m**m**m*	Cell parameters from 649 reflections
*a* = 5.4438 (2) Å	θ = 4.3–40.1°
*c* = 3.11901 (12) Å	µ = 32.61 mm^−^^1^
*V* = 80.05 (1) Å^3^	*T* = 293 K
*Z* = 1	Block, metallic
*F*(000) = 207	0.06 × 0.05 × 0.02 mm

### Samarium trirhodium diboride (III) Data collection

**Table:**

XtaLAB Synergy, Dualflex, HyPix diffractometer	129 independent reflections
Radiation source: micro-focus sealed X-ray tube, PhotonJet (Mo) X-ray Source	128 reflections with *I* > 2σ(*I*)
Mirror monochromator	*R*_int_ = 0.011
Detector resolution: 10.0000 pixels mm^-1^	θ_max_ = 40.1°, θ_min_ = 4.3°
ω scans	*h* = −8→9
Absorption correction: numerical (CrysAlisPro; Rigaku OD, 2021)	*k* = −7→7
*T*_min_ = 0.324, *T*_max_ = 0.542	*l* = −3→5
696 measured reflections	

### Samarium trirhodium diboride (III) Refinement

**Table:**

Refinement on *F*^2^	0 restraints
Least-squares matrix: full	*w* = 1/[σ^2^(*F*_o_^2^) + (0.0213*P*)^2^ + 0.0484*P*] where *P* = (*F*_o_^2^ + 2*F*_c_^2^)/3
*R*[*F*^2^ > 2σ(*F*^2^)] = 0.012	(Δ/σ)_max_ < 0.001
*wR*(*F*^2^) = 0.032	Δρ_max_ = 1.76 e Å^−^^3^
*S* = 1.13	Δρ_min_ = −0.97 e Å^−^^3^
129 reflections	Extinction correction: SHELXL-2016/6 (Sheldrick 2016), Fc^*^=kFc[1+0.001xFc^2^λ^3^/sin(2θ)]^-1/4^
9 parameters	Extinction coefficient: 0.034 (3)

### Samarium trirhodium diboride (III) Special details

**Table:**

Geometry. All esds (except the esd in the dihedral angle between two l.s. planes) are estimated using the full covariance matrix. The cell esds are taken into account individually in the estimation of esds in distances, angles and torsion angles; correlations between esds in cell parameters are only used when they are defined by crystal symmetry. An approximate (isotropic) treatment of cell esds is used for estimating esds involving l.s. planes.

### Samarium trirhodium diboride (III) Fractional atomic coordinates and isotropic or equivalent isotropic displacement parameters (Å^2^)

**Table:**

	*x*	*y*	*z*	*U*_iso_*/*U*_eq_	
Sm1	0.000000	0.000000	0.000000	0.00780 (10)	
Rh1	0.500000	0.000000	0.500000	0.00739 (10)	
B1	0.333333	0.666667	0.000000	0.0091 (7)	

### Samarium trirhodium diboride (III) Atomic displacement parameters (Å^2^)

**Table:**

	*U*^11^	*U*^22^	*U*^33^	*U*^12^	*U*^13^	*U*^23^
Sm1	0.00841 (12)	0.00841 (12)	0.00658 (14)	0.00420 (6)	0.000	0.000
Rh1	0.00502 (11)	0.00389 (13)	0.01287 (14)	0.00194 (6)	0.000	0.000
B1	0.0085 (10)	0.0085 (10)	0.0102 (15)	0.0043 (5)	0.000	0.000

### Samarium trirhodium diboride (III) Geometric parameters (Å, º)

**Table:**

Sm1—Sm1^i^	3.1190 (1)	Sm1—Rh1^x^	3.1370 (1)
Sm1—Sm1^ii^	3.1190 (1)	Rh1—B1^xi^	2.2140 (1)
Sm1—Rh1^iii^	3.1370 (1)	Rh1—B1^xii^	2.2140 (1)
Sm1—Rh1^iv^	3.1370 (1)	Rh1—B1^xiii^	2.2140 (1)
Sm1—Rh1	3.1370 (1)	Rh1—B1^xiv^	2.2140 (1)
Sm1—Rh1^v^	3.1370 (1)	Rh1—Rh1^xv^	2.7219 (1)
Sm1—Rh1^vi^	3.1370 (1)	Rh1—Rh1^xvi^	2.7219 (1)
Sm1—Rh1^vii^	3.1370 (1)	Rh1—Rh1^iii^	2.7219 (1)
Sm1—Rh1^viii^	3.1370 (1)	Rh1—Rh1^xvii^	2.7219 (1)
Sm1—Rh1^ix^	3.1370 (1)	Rh1—Rh1^i^	3.1190 (1)
Sm1—Rh1^ii^	3.1370 (1)	Rh1—Rh1^ii^	3.1190 (1)
			
Sm1^i^—Sm1—Sm1^ii^	180.0	B1^xiii^—Rh1—Rh1^xv^	52.069 (1)
Sm1^i^—Sm1—Rh1^iii^	60.189 (1)	B1^xiv^—Rh1—Rh1^xv^	127.931 (1)
Sm1^ii^—Sm1—Rh1^iii^	119.811 (1)	B1^xi^—Rh1—Rh1^xvi^	127.931 (1)
Sm1^i^—Sm1—Rh1^iv^	119.811 (1)	B1^xii^—Rh1—Rh1^xvi^	52.069 (1)
Sm1^ii^—Sm1—Rh1^iv^	60.189 (1)	B1^xiii^—Rh1—Rh1^xvi^	127.931 (1)
Rh1^iii^—Sm1—Rh1^iv^	180.0	B1^xiv^—Rh1—Rh1^xvi^	52.069 (1)
Sm1^i^—Sm1—Rh1	60.189 (2)	Rh1^xv^—Rh1—Rh1^xvi^	180.0
Sm1^ii^—Sm1—Rh1	119.811 (1)	B1^xi^—Rh1—Rh1^iii^	52.069 (1)
Rh1^iii^—Sm1—Rh1	51.423 (1)	B1^xii^—Rh1—Rh1^iii^	127.931 (1)
Rh1^iv^—Sm1—Rh1	128.6	B1^xiii^—Rh1—Rh1^iii^	52.069 (1)
Sm1^i^—Sm1—Rh1^v^	119.811 (1)	B1^xiv^—Rh1—Rh1^iii^	127.931 (1)
Sm1^ii^—Sm1—Rh1^v^	60.189 (2)	Rh1^xv^—Rh1—Rh1^iii^	60.0
Rh1^iii^—Sm1—Rh1^v^	128.6	Rh1^xvi^—Rh1—Rh1^iii^	120.0
Rh1^iv^—Sm1—Rh1^v^	51.423 (1)	B1^xi^—Rh1—Rh1^xvii^	127.931 (1)
Rh1—Sm1—Rh1^v^	180.0	B1^xii^—Rh1—Rh1^xvii^	52.069 (1)
Sm1^i^—Sm1—Rh1^vi^	60.189 (2)	B1^xiii^—Rh1—Rh1^xvii^	127.931 (1)
Sm1^ii^—Sm1—Rh1^vi^	119.811 (1)	B1^xiv^—Rh1—Rh1^xvii^	52.069 (1)
Rh1^iii^—Sm1—Rh1^vi^	51.423 (1)	Rh1^xv^—Rh1—Rh1^xvii^	120.0
Rh1^iv^—Sm1—Rh1^vi^	128.577 (1)	Rh1^xvi^—Rh1—Rh1^xvii^	60.0
Rh1—Sm1—Rh1^vi^	97.428 (2)	Rh1^iii^—Rh1—Rh1^xvii^	180.0
Rh1^v^—Sm1—Rh1^vi^	82.572 (2)	B1^xi^—Rh1—Rh1^i^	45.219 (1)
Sm1^i^—Sm1—Rh1^vii^	119.811 (1)	B1^xii^—Rh1—Rh1^i^	134.781 (2)
Sm1^ii^—Sm1—Rh1^vii^	60.189 (2)	B1^xiii^—Rh1—Rh1^i^	134.781 (1)
Rh1^iii^—Sm1—Rh1^vii^	128.577 (1)	B1^xiv^—Rh1—Rh1^i^	45.219 (1)
Rh1^iv^—Sm1—Rh1^vii^	51.423 (1)	Rh1^xv^—Rh1—Rh1^i^	90.0
Rh1—Sm1—Rh1^vii^	82.572 (2)	Rh1^xvi^—Rh1—Rh1^i^	90.0
Rh1^v^—Sm1—Rh1^vii^	97.428 (2)	Rh1^iii^—Rh1—Rh1^i^	90.0
Rh1^vi^—Sm1—Rh1^vii^	180.0	Rh1^xvii^—Rh1—Rh1^i^	90.0
Sm1^i^—Sm1—Rh1^viii^	60.189 (1)	B1^xi^—Rh1—Rh1^ii^	134.781 (2)
Sm1^ii^—Sm1—Rh1^viii^	119.811 (1)	B1^xii^—Rh1—Rh1^ii^	45.219 (2)
Rh1^iii^—Sm1—Rh1^viii^	120.379 (3)	B1^xiii^—Rh1—Rh1^ii^	45.219 (1)
Rh1^iv^—Sm1—Rh1^viii^	59.621 (3)	B1^xiv^—Rh1—Rh1^ii^	134.781 (1)
Rh1—Sm1—Rh1^viii^	97.428 (2)	Rh1^xv^—Rh1—Rh1^ii^	90.0
Rh1^v^—Sm1—Rh1^viii^	82.572 (2)	Rh1^xvi^—Rh1—Rh1^ii^	90.0
Rh1^vi^—Sm1—Rh1^viii^	97.428 (1)	Rh1^iii^—Rh1—Rh1^ii^	90.0
Rh1^vii^—Sm1—Rh1^viii^	82.572 (1)	Rh1^xvii^—Rh1—Rh1^ii^	90.0
Sm1^i^—Sm1—Rh1^ix^	119.811 (1)	Rh1^i^—Rh1—Rh1^ii^	180.0
Sm1^ii^—Sm1—Rh1^ix^	60.189 (1)	B1^xi^—Rh1—Sm1	110.498 (2)
Rh1^iii^—Sm1—Rh1^ix^	59.621 (3)	B1^xii^—Rh1—Sm1	69.502 (1)
Rh1^iv^—Sm1—Rh1^ix^	120.379 (3)	B1^xiii^—Rh1—Sm1	69.501 (1)
Rh1—Sm1—Rh1^ix^	82.572 (2)	B1^xiv^—Rh1—Sm1	110.499 (1)
Rh1^v^—Sm1—Rh1^ix^	97.428 (2)	Rh1^xv^—Rh1—Sm1	115.711 (1)
Rh1^vi^—Sm1—Rh1^ix^	82.572 (1)	Rh1^xvi^—Rh1—Sm1	64.3
Rh1^vii^—Sm1—Rh1^ix^	97.428 (1)	Rh1^iii^—Rh1—Sm1	64.289 (1)
Rh1^viii^—Sm1—Rh1^ix^	180.0	Rh1^xvii^—Rh1—Sm1	115.7
Sm1^i^—Sm1—Rh1^ii^	119.811 (1)	Rh1^i^—Rh1—Sm1	119.811 (2)
Sm1^ii^—Sm1—Rh1^ii^	60.189 (1)	Rh1^ii^—Rh1—Sm1	60.189 (2)
Rh1^iii^—Sm1—Rh1^ii^	82.572 (2)	B1^xi^—Rh1—Sm1^xviii^	69.502 (2)
Rh1^iv^—Sm1—Rh1^ii^	97.428 (2)	B1^xii^—Rh1—Sm1^xviii^	110.498 (1)
Rh1—Sm1—Rh1^ii^	59.621 (3)	B1^xiii^—Rh1—Sm1^xviii^	110.499 (1)
Rh1^v^—Sm1—Rh1^ii^	120.379 (4)	B1^xiv^—Rh1—Sm1^xviii^	69.501 (1)
Rh1^vi^—Sm1—Rh1^ii^	128.577 (1)	Rh1^xv^—Rh1—Sm1^xviii^	64.3
Rh1^vii^—Sm1—Rh1^ii^	51.423 (1)	Rh1^xvi^—Rh1—Sm1^xviii^	115.7
Rh1^viii^—Sm1—Rh1^ii^	128.577 (1)	Rh1^iii^—Rh1—Sm1^xviii^	115.7
Rh1^ix^—Sm1—Rh1^ii^	51.423 (1)	Rh1^xvii^—Rh1—Sm1^xviii^	64.3
Sm1^i^—Sm1—Rh1^x^	60.189 (1)	Rh1^i^—Rh1—Sm1^xviii^	60.189 (1)
Sm1^ii^—Sm1—Rh1^x^	119.811 (1)	Rh1^ii^—Rh1—Sm1^xviii^	119.811 (1)
Rh1^iii^—Sm1—Rh1^x^	97.428 (2)	Sm1—Rh1—Sm1^xviii^	180.0
Rh1^iv^—Sm1—Rh1^x^	82.572 (2)	Rh1^xix^—B1—Rh1^xx^	138.425 (1)
Rh1—Sm1—Rh1^x^	120.379 (4)	Rh1^xix^—B1—Rh1^xxi^	75.862 (3)
Rh1^v^—Sm1—Rh1^x^	59.621 (3)	Rh1^xx^—B1—Rh1^xxi^	89.562 (4)
Rh1^vi^—Sm1—Rh1^x^	51.423 (1)	Rh1^xix^—B1—Rh1^vi^	89.562 (4)
Rh1^vii^—Sm1—Rh1^x^	128.577 (1)	Rh1^xx^—B1—Rh1^vi^	75.862 (3)
Rh1^viii^—Sm1—Rh1^x^	51.423 (1)	Rh1^xxi^—B1—Rh1^vi^	138.425 (1)
Rh1^ix^—Sm1—Rh1^x^	128.577 (1)	Rh1^xix^—B1—Rh1^iii^	138.425 (1)
Rh1^ii^—Sm1—Rh1^x^	180.0	Rh1^xx^—B1—Rh1^iii^	75.862 (3)
B1^xi^—Rh1—B1^xii^	180.0	Rh1^xxi^—B1—Rh1^iii^	138.425 (1)
B1^xi^—Rh1—B1^xiii^	89.561 (4)	Rh1^vi^—B1—Rh1^iii^	75.862 (3)
B1^xii^—Rh1—B1^xiii^	90.439 (4)	Rh1^xix^—B1—Rh1^ix^	75.862 (2)
B1^xi^—Rh1—B1^xiv^	90.439 (4)	Rh1^xx^—B1—Rh1^ix^	138.425 (1)
B1^xii^—Rh1—B1^xiv^	89.561 (4)	Rh1^xxi^—B1—Rh1^ix^	75.862 (3)
B1^xiii^—Rh1—B1^xiv^	180.0	Rh1^vi^—B1—Rh1^ix^	138.425 (1)
B1^xi^—Rh1—Rh1^xv^	52.069 (1)	Rh1^iii^—B1—Rh1^ix^	89.562 (4)
B1^xii^—Rh1—Rh1^xv^	127.931 (1)		

Symmetry codes: (i) *x*, *y*, *z*+1; (ii) *x*, *y*, *z*−1; (iii) −*x*+*y*+1, −*x*+1, *z*; (iv) −*x*+*y*, −*x*, *z*−1; (v) *x*−1, *y*, *z*−1; (vi) −*y*, *x*−*y*, *z*; (vii) −*y*, *x*−*y*−1, *z*−1; (viii) −*x*+*y*, −*x*, *z*; (ix) −*x*+*y*+1, −*x*+1, *z*−1; (x) *x*−1, *y*, *z*; (xi) −*x*+1, −*y*+1, −*z*+1; (xii) *x*, *y*−1, *z*; (xiii) −*x*+1, −*y*+1, −*z*; (xiv) *x*, *y*−1, *z*+1; (xv) −*y*+1, *x*−*y*, *z*; (xvi) −*y*, *x*−*y*−1, *z*; (xvii) −*x*+*y*+1, −*x*, *z*; (xviii) *x*+1, *y*, *z*+1; (xix) −*y*, *x*−*y*, *z*−1; (xx) *x*, *y*+1, *z*; (xxi) *x*, *y*+1, *z*−1.
